# Multidrug-Resistant Salmonella Endocarditis of a Native Valve: A Rare Case Presentation

**DOI:** 10.7759/cureus.48396

**Published:** 2023-11-06

**Authors:** Memoona Zahoor, Kamran Ahmad, Musa Kakakhel, Aima Yousaf, Mahnosh Saleh, Mohammad Sayyar, Yasir Ali

**Affiliations:** 1 General Medicine, Lady Reading Hospital, Peshawar, PAK; 2 Internal Medicine, Hayatabad Medical Complex Peshawar, Peshawar, PAK; 3 Internal Medicine, Lady Reading Hospital, Peshawar, PAK; 4 Internal Medicine, Ayub Teaching Hospital, Abbottabad, PAK; 5 Internal Medicine, Khyber Medical College, Peshawar, Peshawar, PAK

**Keywords:** native valve disease, salmonella complications, para enteric salmonella, multidrug resistant (mdr), salmonella typhi endocarditis

## Abstract

*Salmonella* species is a rare cause of infective endocarditis that commonly involves a prosthetic or a previously damaged heart valve. We present a case of a 25-year-old young man with a one and a half month history of cough, fever, shortness of breath, and hemoptysis. Clinical examination revealed bilateral mid-zone crackles, palpable tip of the spleen, and an early diastolic murmur in the aortic (A2) area. Initial laboratory results indicated anemia with leukocytosis, raised inflammatory markers, and low serum albumin. Blood cultures showed the growth of multidrug-resistant *Salmonella typhi*. A radiological workup showed multiple aortic valve vegetation.

*Salmonella* endocarditis was diagnosed based on Duke’s criteria. The patient was treated with culture-sensitive antibiotics and subsequently showed significant clinical recovery. This case highlights a rare multidrug-resistant *Salmonella* endocarditis of a native valve. It also emphasizes the difficulties in making a diagnosis and the benefit of using a multidisciplinary strategy to manage challenging clinical manifestations.

## Introduction

Infective endocarditis (IE) is a cardiac endothelial infection with an annual incidence of 3-10/100,000 people [1]. *Salmonella* species are responsible for more than 1.2 million illnesses each year [2]. It typically presents with mild gastroenteritis but can be more severe, especially in infants, the elderly, and immunocompromised hosts [3]. The most prevalent cause of IE in most studies is Staphylococcus aureus (∼26.6%), followed by viridans group streptococci (18.7%), other streptococci (17.5%), and enterococci (10.5%). These organisms are responsible for 80-90% of all endocarditis cases [4]. *Salmonella* species was discovered to be a rare cause of IE. Furthermore, previous studies have focused more on non-typhoidal infections, owing to their increased relative prevalence [5]. Patients with an underlying cardiac pathology, most notably rheumatic heart disease or prosthetic valve replacement, have been involved in the bulk of documented *Salmonella* cases with cardiac involvement. Additionally, studies have indicated that the mitral valve is the valve that is most likely to be impacted [5].

If not treated properly, it carries a high mortality rate. This emphasizes the importance of selecting an appropriate antibiotic regimen with timely administration of antibiotics to achieve a cure for this disease [6]. Previously reported cases of *Salmonella* endocarditis have been traditionally treated with fluoroquinolones and third-generation cephalosporins. Studies on *Salmonella* endocarditis due to multidrug-resistant *Salmonella typhi* are scarce. Here, we present a rare case of IE, with an atypical presentation due to multidrug-resistant *Salmonella typhi*.

## Case presentation

A 25-year-old male of South Asian descent with no previous comorbidities, working in the tuberculosis (TB) center as a porter, presented with a cough for 45 days, fever for 30 days, shortness of breath for 30 days, and hemoptysis for three days. The cough was associated with sputum initially whitish in color, which later changed into red color. The fever was of high grade (102-104℉) and associated with rigors, chills, and night sweats, which were relieved with over-the-counter medications. There was shortness of breath initially with exertion, which later worsened. The past medical and family history was unremarkable. The patient was an ex-smoker with 5-pack years.

Upon examination, the patient was active, alert, and oriented. He was stable with a pulse of 76 beats/minute, blood pressure of 110/70 mm Hg, and SpO2 of 95% on room air. On chest examination, there was an equal expansion on bilateral palpation, dullness to percussion in the right mid-zone, auscultation of the bilateral upper zone revealing wheezes, bilateral mid-zone crackles, soft and non-tender abdomen, and a palpable spleen tip. Cardiovascular examination revealed an early diastolic murmur in the aortic (A2) area.

His detailed workup to identify the underlying cause consisted of a baseline investigation that included a chest X-ray, sputum acid-fast bacillus (AFB) culture, Gene Xpert test, blood culture, echocardiography, high-resolution CT (HRCT) scan, and inflammatory markers. After sending a blood culture and other investigations, the patient was started empirically on antibiotics according to the local guidelines. thus; ceftriaxone 2 gm (twice a day [BID]) and meropenem 2 gm (BID) were started.

The sputum AFB and Gene Xpert tests were negative for AFB, thus excluding pulmonary TB, which was our initial suspected diagnosis. The rest of the labs are presented in Table 1.

**Table 1:** Basic laboratory investigations and inflammatory markers

Parameter	On Admission	After 48 hours	On discharge	Reference ranges
Complete blood count				
White blood cells	20.4 x 10^3/^uL	15.4 x 10^3^/uL	12.4 x 10^3^/uL	4–11 x 10^3^/uL
Red blood cells	3.92 x 10^6^/uL	3.75 x 10^6^/uL	3.62 x 10^6^/uL	4–6 10^6^/UL
Hemoglobin	10.9 g/dL	9.92 g/dL	10.8 g/dL	11.5–17.5 g/dL
Hematocrit	35.3 %	33.7%	35.9%	36–54%
Platelets count	521 x 10^3^/uL	411 x 10^3^/uL	299 x 10^3^/uL	150–450 x 10^3^/uL
% Neutrophils	88	82	70	40–70
% Lymphocytes	10	15	20	20–45
% Monocytes	1	3	8	0.9–5.2
Inflammatory markers				
C-reactive protein (mg/dL)	11.6	6.4	3.5	<1.0
Erythrocyte sedimentation rate (mm/hour)	110	122	105	0–15
Lactate dehydrogenase (U/L)	303		297	240-280
Procalcitonin (ng/mL)	0.72		0.6	<0.5
Serum electrolytes				
Sodium (mmol/L)	130.4	136	141	135–150
Potassium (mmol/L)	4.78	4.39	4.21	3.5– 5.1
Chloride_ (_mmol/L)	99.4	97	102,3	96–112
Liver function test				
Total bilirubin (mg/dL)	0.6	0.5	0.52	0.1–1.0
Alkaline transferase (U/L)	160	532	302	10–50
Serum albumin (g/dL)	2.6	2.9	3.3	3.5–5.5
Renal function tests				
Urea (mg/dL)	36.36	64.76	59.28	18–45
Creatinine (mg/dL)	0.81	1.04	1.12	0.64–1.2
Uric acid (mg/dL)	4.6		5.2	3.4–7.0

**Figure 1:**
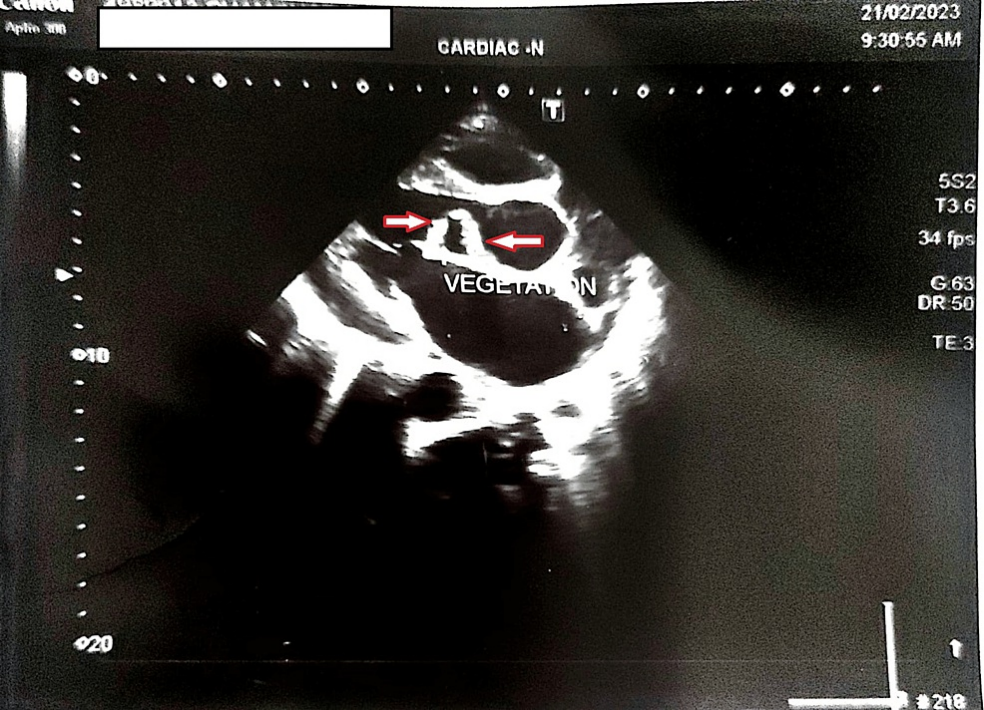
Echocardiogram showing multiple small aortic valve vegetations Red arrows indicate small multiple aortic vegetations

On echocardiogram, there were multiple aortic valve vegetation, severe aortic regurgitation (non-co-optative aortic valve), and moderate mitral regurgitation. Vegetation is shown in Figure 1.

**Figure 2:**
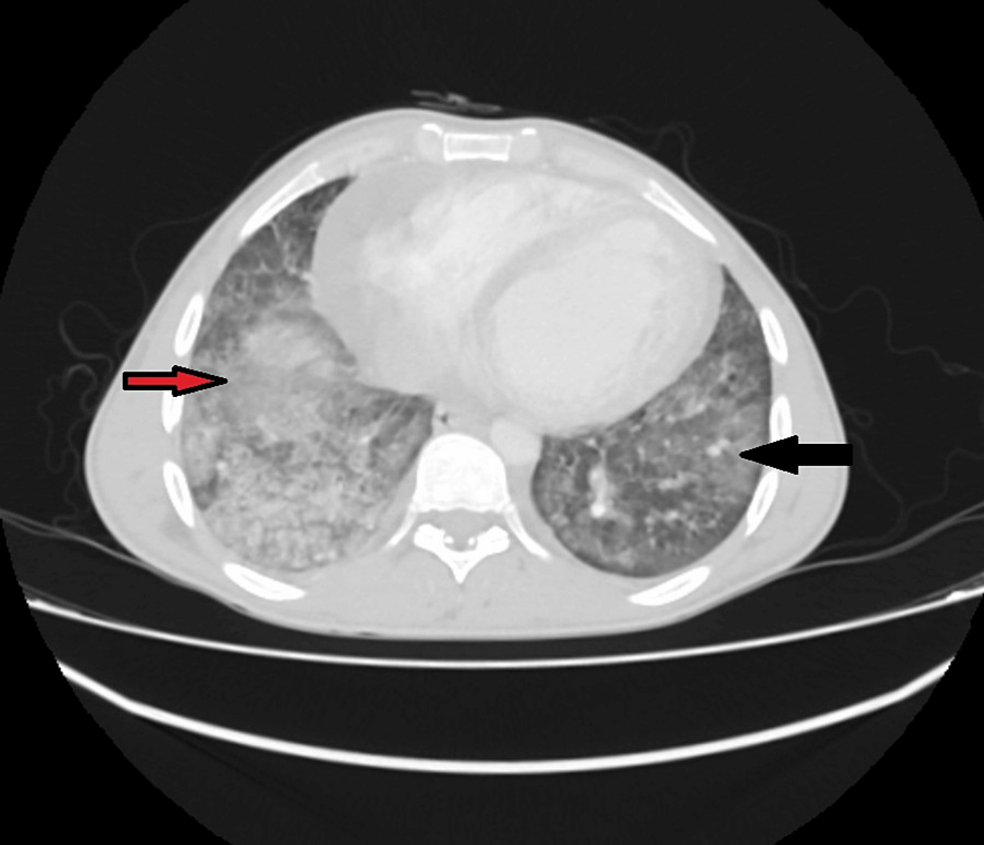
High-resolution CT scan showing bilateral dense multi-lobular shadowing on the background of ground-glass opacification with interlobular and intra-lobular septal thickening. Black arrow indicates ground-glass opacities. Red arrow indicates consolidation in the background of -round glass opacities.

**Figure 3:**
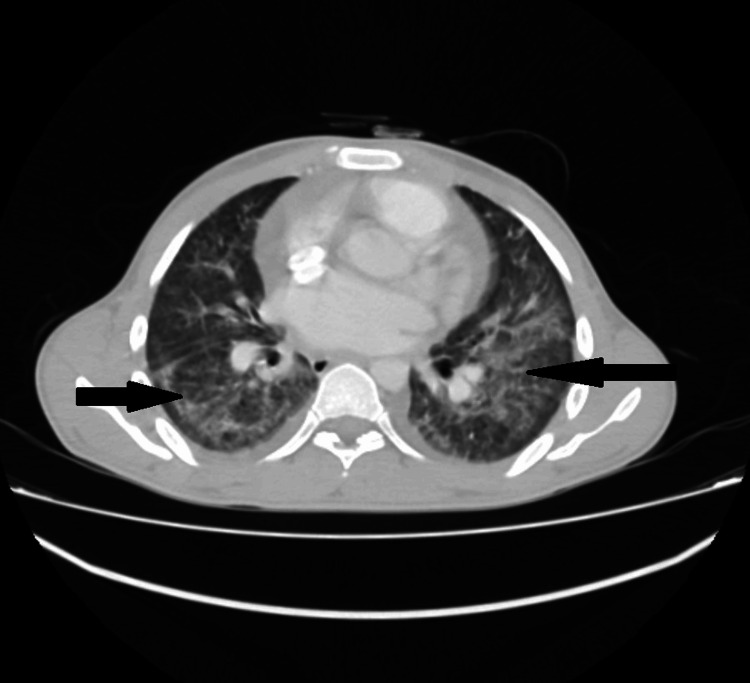
High-resolution CT scan showing bilateral ground-glass opacification, small cavitations, and multiple sub-centimeter mediastinal lymph nodes. Black arrows indicate ground-glass opacities

Chest HRCT showed bilateral dense multi-lobular shadowing on the background of ground-glass opacification with interlobular and intra-lobular septal thickening and multiple sub-centimeter mediastinal lymph nodes, suggestive of an infectious disease process, as shown in Figures 2, 3.

After seven days, the blood culture via the BACTEC 9240 radiometric method was positive for heavy growth of *Salmonella typhi*, which was sensitive to imipenem, meropenem, ceftriaxone, and cefixime, and resistant to ciprofloxacin, co-trimoxazole, chloramphenicol, and ampicillin, and intermediate to azithromycin; therefore, it can be defined as multidrug-resistant as they are resistant to chloramphenicol, ampicillin, and trimethoprim-sulfamethoxazole. By applying Duke’s criteria for IE, the patient fulfilled two major criteria (positive blood culture and vegetation on echocardiography) and two minor criteria (temperature > 38°C and embolic phenomenon); therefore, a diagnosis of definitive endocarditis was made as it fulfilled two major criteria.

He continued the same treatment with ceftriaxone (2 gm BID) and meropenem (2 gm BID) for two weeks. During this period, the patient showed significant clinical recovery and was counselled about further treatment and prevention in special circumstances, such as dental and surgical procedures. He is currently healthy and regularly attends follow-ups at the outpatient department.

## Discussion

*Salmonella* endocarditis is one of the rarest causes of IE worldwide [5]. In studies conducted from 1976 to date, fewer than 90 cases have been reported in the literature, mainly comprising case reports and case series. The total burden of *Salmonella* endocarditis is 0.01-2.9% of all bacterial endocarditis cases. Most often, the affected population is in their sixth decade of life, with the most common valve affecting the mitral valve [5,7].

In most reported cases, the patient had either a previously damaged prosthetic valve or prosthetic valve replacement. Contrary to the reported literature, our patient was in the third decade of life and had native valve *Salmonella* endocarditis of the aortic valve, which is a rare presentation of a rare etiology of bacterial endocarditis.

Unfortunately, *Salmonella *endocarditis is a fatal condition, with mortality ranging from 42% to 69% [5,7-10]. Our patient, however, responded well to medical therapy and survived the event to complete resolution with only medical therapy directed by the culture and sensitivity report.

*Salmonella* infections most often present with gastrointestinal symptoms; however, extra-intestinal manifestations are possible [7]. The optimal treatment of *Salmonella enterica* is clearly defined and subject to the culture and sensitivity of the bacteria involved, drastically changing from country to country. Therefore, local guidelines based on AntiBiogram reports are ideal for consulting when initiating empirical therapy; however, the treatment for valvular or mural endocarditis as an extraintestinal manifestation of *Salmonella *endocarditis is not yet defined, and thus we started our patient empirically on meropenem 2 g BID and ceftriaxone 2 g BID as the burden of extensively drug-resistant *Salmonella* is high in our community. Fernandez et al. recommended the use of potent antimicrobial treatment with or without surgical intervention to address cardiovascular complications [8].

*Salmonella choleraesuis*, *Salmonella typhimurium*, and *Salmonella enteritidis *are commonly known serotypes among the cases, while *Salmonella Thompson* and *Salmonella derby *types have also been reported [9,11,12,13]. Infection with multidrug resistance is associated with a dismal prognosis; however, our patient recovered without incident, as did the case reported by Khan et al. [9].

*Salmonella* endocarditis may be a rare etiology, but in the context of multidrug resistance and extreme drug-resistant pathogens that are prevalent in our province, it should be a cause for concern as virulent strains like the one reported have the capacity to infect native valves and could wreak havoc on the already existing and growing number of patients with previously damaged valves and those with prosthetic valves due to rheumatic fever. Therefore, clinicians should be vigilant when treating *Salmonella* infections.

This case presented with initial ambiguity in diagnosis because of the patient’s significant exposure to TB. Hence this 25-year-old young man with symptoms of fever, cough, and hemoptysis was initially investigated for pulmonary TB. Further workup revealed native valve *Salmonella* IE, confirmed by echo and positive blood cultures. The patient responded well to the empirical antibiotic regimen according to local guidelines, that is, a combination of ceftriaxone and meropenem. The blood culture report later on showed sensitivity to the same regimen. While the patient presented with an unusual native valve endocarditis, the blood cultures revealed drug-resistant bacteria of *Salmonella typhi*.

## Conclusions

In conclusion, this case report underscores several critical points regarding *Salmonella* endocarditis. First and foremost, it highlights the alarming trend of *Salmonella* strains exhibiting extreme drug resistance, posing a formidable challenge in clinical management. Furthermore, the involvement of the native valve, particularly the aortic valve, instead of the more commonly affected mitral valve, accentuates the variability of this condition and the need for a comprehensive diagnostic approach. Equally important, early detection of complications arising from *Salmonella* infection, such as endocarditis, assumes paramount significance. Therefore, our findings underscore the necessity for heightened vigilance in screening patients for such complications, enabling timely intervention and better outcomes in the management of *Salmonella*-related endocarditis. This case serves as a reminder of the evolving clinical landscape of *Salmonella* infections and the importance of tailored and vigilant clinical strategies.
